# Effect of Flowable Thrombin-Containing Collagen-Based Hemostatic Matrix for Preventing Pancreatic Fistula after Pancreatectomy: A Randomized Clinical Trial

**DOI:** 10.3390/jcm9103085

**Published:** 2020-09-24

**Authors:** Yejong Park, Jae Hyung Ko, Dae Ryong Kang, Jun Hyeok Lee, Dae Wook Hwang, Jae Hoon Lee, Woohyung Lee, Jaewoo Kwon, Si-Nae Park, Ki-Byung Song, Song Cheol Kim

**Affiliations:** 1 Division of Hepato-Biliary Pancreatic Surgery, Department of Surgery, University of Ulsan College of Medicine & Asan Medical Center, 88, Olympic-ro 43-gil, Songpa-gu, Seoul 05505, Korea; blackpig856@gmail.com (Y.P.); dwhwang@amc.seoul.kr (D.W.H.); gooddr23@naver.com (J.H.L.); ywhnet@gmail.com (W.L.); skunlvup@naver.com (J.K.); 2 Regenerative Medicine Research Center, Dalim Tissen Co., Ltd., 31, Yeonhui-ro, Mapo-gu, Seoul 05505, Korea; rnd10@dalimtissen.com; 3 Department of Precision Medicine, Wonju College of Medicine, Yonsei University, 1 Yonseidae-gil, Wonju, Gangwon-do 26493, Korea; dr.kang@yonsei.ac.kr (D.R.K.); ljh0101@yonsei.ac.kr (J.H.L.); 4 Department of Biostatistics, Wonju College of Medicine, Yonsei University, 1 Yonseidae-gil, Wonju, Gangwon-do 26493, Korea; 5 Asan Medical Institute of Convergence Science and Technology (AMIST), 88, Olympic-ro 43-gil, Songpa-gu, Seoul 05505, Korea

**Keywords:** pancreatic fistula, pancreatectomy, pancreatic neoplasm

## Abstract

Background: The aim of this study was to evaluate the safety and efficacy of a flowable hemostatic matrix, and their effects for postoperative pancreatic fistula (POPF) after pancreatectomy. Methods: This was a randomized, clinical, single-center, single-blind (participant), non-inferiority, phase IV, and parallel-group trial. The primary endpoint was the incidence of POPF. The secondary endpoints were risk factors for POPF, drain removal days, incidence of complication, 90-day mortality, and length of hospital stay. Results: This study evaluated a total of 53 patients, of whom 26 patients were in the intervention group (flowable hemostatic matrix) and 27 patients were in the control group (thrombin-coated collagen patch). POPF was more common in the control group than in the intervention group (59.3% vs. 30.8%, *p* = 0.037). Among participants who underwent distal pancreatectomy, POPF (33.3% vs. 92.3%, *p* = 0.004), and clinically relevant POPF (8.3% vs. 46.2%, *p* = 0.027) was more common in the control group. A multivariate logistic regression model identified flowable hemostatic matrix use as an independent negative risk factor for POPF, especially in cases of distal pancreatectomy (DP) (odds ratio 17.379, 95% confidential interval 1.453–207.870, *p* = 0.024). Conclusion: Flowable hemostatic matrix application is a simple, feasible, and effective method of preventing POPF after pancreatectomy, especially for patients with DP. Non-inferiority was demonstrated in the efficacy of preventing POPF in the intervention group compared to the control group.

## 1. Introduction

Pancreatectomy including pancreaticoduodenectomy (PD) and distal pancreatectomy (DP) are standard surgical procedures in cases of pancreatic neoplasms, respectively [1,2,3]. However, the morbidity of this procedure is still high, ranging from 30% to 40% for PD [1,4]. The complication rate of PD is higher than that of other operations, and the high morbidity is mainly attributed to the occurrence of postoperative pancreatic fistula (POPF). In addition, POPF remains the leading cause of morbidity after DP, with a frequency ranging from 13% to 64% [3,5,6]. The clinically significant complication after pancreatectomy is POPF, which can lead to secondary complications such as intra-abdominal abscess, sepsis, and bleeding.

Despite attempts at reducing the incidence of POPF, which include pancreaticoenteric anastomosis, use of fibrin sealants, pancreatic stent insertion, and administration of octreotide, the incidence of POPF after PD has not considerably decreased. In addition, there are no validated recommendations or guidelines for the closure of the pancreatic remnant after DP, and no consensus exists on an optimal method for closure of the pancreatic stump [7,8]. The use of several different methods to secure the pancreatic remnant, including duct ligation, ultrasonic dissection, fibrin glue, patches and meshes, pancreaticoenteric anastomosis, and handsewn and stapler closure, possibly with bovine pericardial buttress, demonstrates the ongoing controversy [9,10,11].

Collagen has low antigenicity, hemostatic effects, and cell adhesion ability, and it is commonly used as a major component of hemostatic agents and artificial tissue substitutes [7,12,13,14,15,16,17,18,19]. In addition, collagen provides an environment in which fibroblasts can proliferate and induces wound healing by inactivating elastase and matrix metalloproteinases (MMPs) [20,21,22]. The prevention of POPF by applying collagen-based fibrin sealant patches to the anastomosis site or pancreatic stump has been reported previously. However, the usefulness of using fibrin sealant patches at the pancreatectomy site is still unclear [7,12,13,14,15,16,17,23].

CollaStat^®^ (Dalim Tissen Co. Ltd., Seoul, Korea) is a novel flowable hemostatic agent that combines a collagen matrix with thrombin, a paste-like matrix that exhibits both passive and active mechanisms of actions, which are similar to FloSeal^®^ (Baxter Healthcare, Deerfield, IL, USA) [18,19]. To the best of our knowledge, no studies have evaluated the efficacy of a flowable hemostatic matrix for the prevention of POPF. The aim of this study was to evaluate the safety and efficacy of a flowable hemostatic agent compared with a thrombin-coated collagen patch (CollaSeal^®^, Dalim Tissen Co. Ltd., Seoul, Korea) and their effect on clinical outcomes including POPF in a randomized controlled clinical trial.

## 2. Methods

### 2.1. Trial Design

We enrolled patients who underwent pancreatectomy in the Division of Hepato-Biliary and Pancreatic Surgery of the Department of Surgery at Asan Medical Center between February 2018 and September 2018. This was a randomized, clinical, single-center, single-blind (participant), non-inferiority, phase IV, and parallel-group trial. The study complied with the Declaration of Helsinki and was approved and overseen by the institutional review board (Number: 2017-1062) of Asan Medical Center. This study was registered at clinicaltrials.gov (NCT04357483) and performed according to CONSORT guidelines [24].

### 2.2. Inclusion and Exclusion Criteria

Patients were included if they (1) were 20–80 years on the day of enrollment; (2) had Eastern Cooperative Oncology Group (ECOG) performance scores of 0–2; (3) had potentially curable benign, premalignant, or malignant pancreatic disease, as shown by preoperative imaging (computed tomography, magnetic resonance imaging, and/or positron emission tomography); (4) had appropriate bone marrow function (WBC count of at least 3000/mm^3^, platelet count of at least 100,000/mm^3^); (5) had appropriate liver function (AST/ALT less than 3 times the upper limit of normal); (6) had appropriate renal function (creatinine level greater than 1.5 times the upper limit of normal); (7) provided written informed consent. Patients were excluded if they (1) had active or uncontrolled infections; (2) had severe psychiatric or neurological disorders; (3) had alcohol or other drug addictions; (4) were included in other clinical studies that may affect this study; (5) had uncontrolled heart disease; (6) had moderate or severe comorbidities that are thought to have affected the quality of life or nutritional status; (7) had pelvic tumors, benign tumors, or malignant tumors in other organs; (8) were pregnant or planning on becoming pregnant during the follow-up period; (9) had lymphatic or coagulation disease; or (10) had known sensitivity or allergy to bovine and/or porcine substance(s).

### 2.3. Surgical Technique and Study Protocol

The procedures for PD and DP in our institution have been reported previously [4,6,17,25,26]. Furthermore, the detailed description of pancreaticojejunostomy (PJ) during PD is in detail in our previous study [17]. PJ was carried out using the double-layered, end-to-side duct-to-mucosa method. In addition, when left-sided pancreatectomy was performed, to transect the pancreas safely in both the open and laparoscopic procedures, straight or rotated endoscopic linear staplers of various sizes (staple height, 3.5 to 4.2 mm) were used depending on the thickness or hardness of the pancreas. After transecting the pancreas, 4 or 5 small titanium clips were applied along the stapling line to prevent pancreatic fistula and bleeding from the resected stump.

Before closure, a 1–3 closed suction drain was inserted into the bed of the removed portion of the pancreas and maintained for at least 3 days postoperatively to prevent intra-abdominal fluid collection and identify POPF. Each patient was allowed sips of water on postoperative day (POD) 1 and a soft blended diet on POD 2. Postoperative assessment included repeated measurements of amylase concentrations in the serum and drainage fluid while the drain was in place. POPF was defined as a drain fluid amylase concentration greater than 3 times the upper normal serum concentration after POD 3 as defined by the International Study Group on Pancreatic Surgery (ISGPS) criteria [27].

### 2.4. Application of Thrombin-Containing Collagen Hemostatic Matrix (T-C Matrix) in the Intervention Group

**Figure 1:**
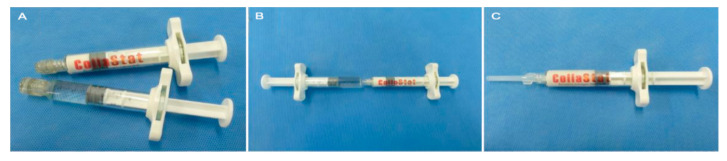
Preparation of CollaStat^®^. (**A**) CollaStat consists of two syringes: one for collagen granules and thrombin and the other for CaCl_2_ solution. (**B**) CollaStat^®^ can be easily prepared by connecting the two syringes and mixing them. (**C**) After mixing and detaching the CaCl_2_ syringe, CollaStat is ready for use by connecting the enclosed application tip.

The T-C matrix (CollaStat^®^; Dalim Tissen Co. Ltd., Seoul, Korea) is a flowable collagen-based hemostatic matrix [18,19]. The T-C matrix is comprised of two connectable syringes, syringe A and syringe B. Syringe A contains porcine skin-derived atelocollagen, thrombin. Syringe B contains calcium chloride solution (Figure 1). The collagen granules of T-C matrix are made from highly purified type I atelocollagen derived from the porcine dermis, which shows biocompatibility due to the minimally immunogenic, biocompatible, and biodegradable properties of atelocollagen. The matrix can be prepared after mixing the materials in the syringes. In the intervention group, T-C matrix was applied to the anastomosis site and pancreatic stump after PJ or DP. The matrix was approved for use by the Korean Food and Drug Administration.

### 2.5. Application of Thrombin-Coated L-Dopa-Containing Collagen Patch (T-CD Patch) in the Control Group

For patients in the active control group, a 5.0 cm × 5.0 cm T-CD patch (CollaSeal^®^; Dalim Tissen Co. Ltd., Seoul, Korea) was applied to the front and back of the anastomosis site. The T-CD patch, which has been clinically used for the prevention of POPF and postpancreatectomy hemorrhage (PPH), is a sponge-like wound dressing incorporated with thrombin and L-DOPA. Owing to thrombin and L-DOPA, a T-CD patch can effectively accomplish hemostasis and adhere to the wound site. In addition, a T-CD patch has a honeycomb-like porous structure, which contributes to a good absorption capacity for blood or exudates.

### 2.6. Outcome Measures

The primary endpoint of this study was the incidence of POPF. The evaluation of pancreatic fistula was based on the ISGPS criteria [27]. According to the criteria, POPF was defined as a drain fluid amylase concentration greater than 3 times the upper normal serum concentration after POD 3. The secondary endpoints were risk factors for POPF, drain removal days, incidence of complication according to the Clavien–Dindo classification [28], 90-day mortality, and length of hospital stay.

### 2.7. Sample Size

The POPF prevention rate of fibrin sealants has been reported as 88% previously [7]. The non-inferiority limit was calculated to be 0.22 based on the case where more than 75% of 88% of existing treatments were confirmed. When the lower limit of the 95% CI for the difference between the two groups was greater than −0.22, it was judged that TC-matrix was not inferior to T-CD patch. Based on this hypothesis, a sample size of 54 patients (27 in each group) was estimated based on type 1 error α = 0.05 and power (1 − β) = 0.8 using a two-sided χ^2^ test. Factoring in a 10% dropout rate, we recruited 60 patients (30 per group).

### 2.8. Randomization

A total of 58 patients were randomized with block randomization before surgery. We performed block randomization to correct the imbalance between the intervention and control groups. We assigned A to the intervention group and the groups were determined as follows: (1) ABBA, (2) BBAA, (3) ABAB, (4) BABA, (5) AABB, and (6) BAAB. We selected blocks and allocated surgical procedures based on the number of a die rolled once. Independent researchers randomized patients for this study.

### 2.9. Statistical Analysis

The results are presented as the mean with standard deviation (SD) or median with interquartile range (IQR). Patient demographics and clinical characteristics were compared using the χ^2^ test or Fisher’s exact test for categorical variables and the Student’s *t*-test and Mann–Whitney test for continuous variables. In assessing the risk factors associated with overall POPF and clinically relevant POPF, only variables statistically significant in univariate analysis were included in multivariate analysis, which was performed using logistic regression. All statistical analyses were performed using SPSS version 21.0 (IBM Corp., Armonk, NY, USA) with p values less than 0.05 considered statistically significant.

## 3. Results

**Figure 2:**
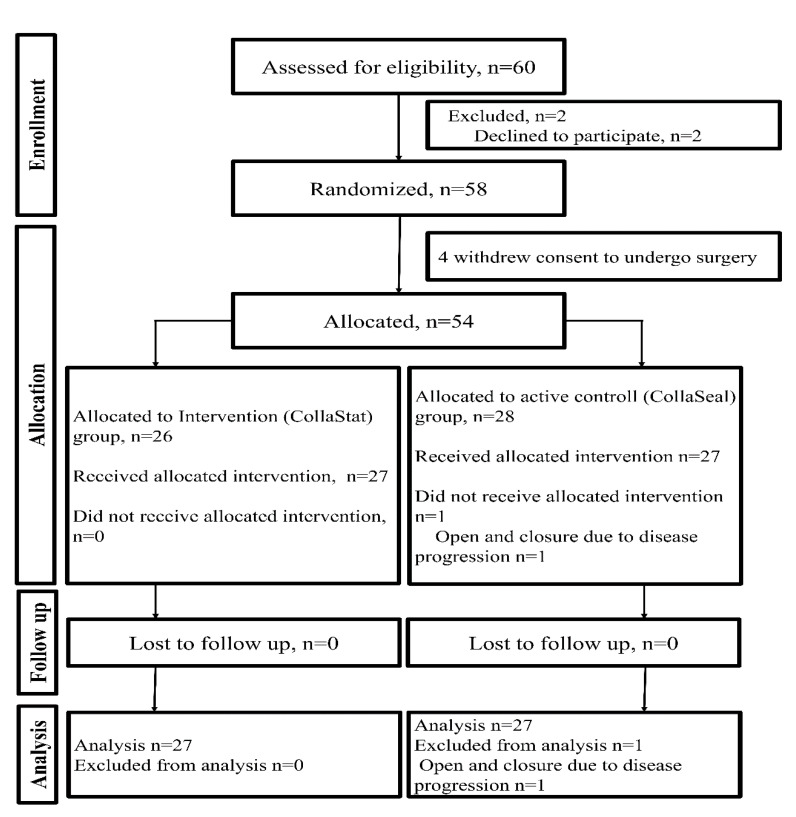
CONSORT flow diagram of the trial.

Participants were recruited from February 2018 to September 2018 and followed up until February 2019. A total of 60 patients were enrolled; however, 2 patients declined to participate. Of the 58 randomized patients, 4 patients had to withdraw consent to undergo surgery, and the remaining 54 patients were allocated to two groups (intervention group: *n* = 26, active control group: *n* = 28). One patient in the control group did not undergo pancreatectomy due to the progression of pancreatic cancer; thus, this participant was excluded from further analysis. Therefore, this study evaluated a total of 53 patients, with 26 patients in the intervention group and 27 patients in the control group (Figure 2).

Age, sex, Charlson comorbidity score, operative type, operative name, additional organ resection, operative time, estimated blood loss, pathological diagnosis, pancreatic texture, mass size, pancreatic duct size, alternative fistula risk score, and neoadjuvant chemotherapy were not statistically different between the two groups (Table 1).

### 3.1. Primary Outcomes

POPF was more common in the active control group than in the intervention group (59.3% vs. 30.8%, *p* = 0.037; Table 2). However, there was no statistical difference in clinically relevant POPF between the two groups (15.4% vs. 29.6%, *p* = 0.409).

As a result of evaluating the POPF prevention rate in this study, it was 60.2% (18/26 patients) for the intervention group and 40.7% (11/27 patients) for the control group. The upper limit of the 95% confidence interval for the difference in the POPF prevention rate between the two groups was −2.83%, which was less than the non-inferiority margin of 22%.

### 3.2. Secondary Outcomes

The postoperative outcomes of all patients are shown in Table 2. There were no significant differences in postoperative outcomes except for the occurrence of POPF (Table 2). The median length of hospital stay (8 days vs. 9 days, *p* = 0.284) and median drain removal days (4 days vs. 5 days, *p* = 0.241) were not significantly different between the two groups. In addition, the complication rate was significantly different (34.6% vs. 63.0%, *p* = 0.039). By limiting the Clavien–Dindo classification to ≥grade 3, there was no significant difference in the complication grade between the intervention and control groups (19.2% vs. 11.1%, *p* = 0.444). There was no difference in the 90-day mortality between the two groups.

There were 4 patients with clinically relevant POPF in the intervention group, and all of them had grade B POPF. Among these patients, 1 patient underwent antibiotic therapy, and 2 patients underwent endoscopic ultrasound-guided gastrocystostomy for intra-abdominal complicated fluid collection. The other patient underwent embolization for pseudoaneurysm of the gastroduodenal artery stump. In the control group, there were 8 patients with clinically relevant POPF, and 1 of them had grade C POPF (this patient underwent reoperation for surgical site infection). A total of 6 patients underwent antibiotic therapy, and 1 patient underwent anticoagulation therapy for portal vein thrombosis. Red blood cell (RBC) transfusions were made only to 2 patients in each group. The amount of RBC was the same as 720 ± 113.14 mL in both groups. The readmission rate (19.2% vs. 11.1%, *p* = 0.409) was not different between the two groups.

### 3.3. Sub-Analysis of Patients Who Underwent Distal Pancreatectomy

Among patients who underwent DP, POPF (33.3% vs. 92.3%, *p* = 0.004) and clinically relevant POPF (8.3% vs. 46.2%, *p* = 0.027) were more common in the control group than in the intervention group. There were no statistically significant differences in drain removal days, length of hospital stay, 90-day mortality, readmission rate, and complication rate. Furthermore, the complication rate was significantly different (33.3% vs. 92.3%, *p* = 0.004). By limiting the Clavien–Dindo classification to ≥ grade 3, there was no significant difference in the complication grade between the intervention and control groups (8.3% vs. 23.1%, *p* = 0.593) (Table 3).

The POPF rate was 28.6% (*p* > 0.999) in both the intervention and control groups, showing no statistically significant difference. In addition, CR-POPF (21.4% vs. 14.3%, *p* > 0.999), postoperative complication (35.7% vs. 35.7%, *p* > 0.999), complication ≥ Grade III (21.4% vs. 0.0%, *p* = 0.119), median length of hospital stay (10 days vs. 9 days, *p* > 0.999), and readmission rate (28.6% vs. 14.3%, *p* = 0.648) showed no difference between the two groups.

### 3.4. Risk Factors for POPF and Clinically Relevant POPF

A multivariate logistic regression model identified T-C matrix use (OR 4.744, 95% CI 1.172–19.210, *p* = 0.029), pancreatic duct ≥ 3 mm (OR 7.120, 95% CI 1.399–36.241, p = 0.018), and form or hard pancreatic texture (OR 6.525, 95% CI 1.668–25.529, *p* = 0.007) as independent negative risk factors for POPF in the current study (Table 4). In addition, multivariate logistic regression analysis of clinically relevant POPF identified soft pancreatic as an independent risk factor (OR: 7.353, 95% CI: 1.429~37.847, *p* = 0.017).

T-C matrix application was a negative risk factor for POPF (Table 4) and clinically relevant POPF (OR: 10.286, 95% CI: 1.018~103.948, *p* = 0.048) among patients who underwent left-sided pancreatectomy.

## 4. Discussion

This prospective study showed that applying a T-C matrix to the PJ or pancreatic stump after pancreatectomy significantly reduced the incidence of POPF compared with that in the active control group (T-CD patch). POPF was more common in the control group than in the intervention group (59.3% vs. 30.8%, *p* = 0.037; Table 2). In a multivariate logistic regression model, T-C matrix application was a negative risk factor for POPF (Table 4), especially among patients who underwent DP.

To the best of our knowledge, no studies have evaluated the efficacy of a flowable hemostatic matrix for the prevention of POPF. Two retrospective studies reported that fibrin sealant patches are feasible and safe with 7.4%–20% POPF rates after PD with pancreaticojejunostomy [13,29]. Schindl et al. [30] conducted a multicenter, randomized clinical trial to investigate the effect of using thrombin-coated collagen patches after PD. In the study, the rates of POPF were 63% in the intervention group and 56% in the control group, and clinically relevant POPF rates were 23% in the intervention group and 14% in the control group. The study reported that there was no POPF reduction with the use of thrombin-coated collagen patches after PD. Similarly, there was no POPF reduction in a prospective study of patients who underwent PD in our center [17]. The POPF rate was 25.8% in the intervention group and 37.1 in the control group (*p* = 0.246). In the current study, we attempted to evaluate the safety and efficacy of a flowable hemostatic agent compared with a thrombin-coated collagen patch and their effect on clinical outcomes including POPF. The POPF rate was 28.6% among patients who underwent PD in the control group (thrombin-coated collagen patch) and was 28.6% among patients who underwent PD in the intervention group (flowable hemostatic matrix); thus, there was no POPF reduction effect. Clinically relevant POPF rates were 21.4% in the intervention group and 14.3% in the control group (p > 0.999) among the patients who underwent PD.

Several studies have been conducted on the use of thrombin-coated collagen patches for preventing POPF after DP. Silvestri et al. [14] reported that the use of fibrin sealant patches appeared to be associated with a lower incidence of grade C POPF. However, the POPF rate was not different in both groups (intervention: 36.1% vs. control: 41.6%, *p* = n.s). Two previous multicenter, randomized controlled trials reported that there was no significant effect on the rate of POPF after DP. Montorsi et al. [7] reported POPF rates of 62% and 68% in the intervention and control groups, respectively (*p* = 0.267), and Sa Cunha et al. [31] reported rates of 54.5% and 56.6%, respectively (*p* = 0.807). There was no statistically significant difference in clinically relevant POPF. In another randomized trial, the POPF rate was reported as 70.8% in the intervention group and 54.7% in the control group [10]. The study indicated there are no clinically relevant benefits in applying a patch in terms of reducing the incidence and severity of POPF after DP. In the current study, the POPF rate was reduced among patients who underwent DP, and a low incidence of clinically relevant POPF was observed when a flowable hemostatic matrix was applied (Table 3). Moreover, in a multivariate logistic regression model, T-C matrix application was a negative risk factor for POPF, especially among patients who underwent DP (Table 4).

In this study, the overall incidence of POPF, especially after DP, was 64.0%, and the rate of clinically relevant POPF was 28.0%, which was greater than that reported in previous studies [7,14,31]. As mentioned previously [10], this may be explained by the rigid application of the ISGPS criteria by an independent research coordinator and not the doctors who were involved in this study. In addition, several studies omitted grade A (or BL) fistula from the analyses because intra-abdominal drains were not routinely used during surgery [32,33]. Similar to previous studies [7,31], most of the POPF cases were biochemical leakage (*n* = 12, 50%) in this study.

A T-C matrix is a novel flowable collagen-based hemostatic agent. The matrix can be prepared via the following simple steps: (1) connecting two syringes, (2) mixing the contents, and (3) application of the mixed matrix on the defect site. This simple procedure allows the preparation of a hemostatic matrix without a time-consuming thrombin re-constitution process. In addition, flowable hemostatic agents may be more advantageous than non-flowable ones as they can cover irregular wound surfaces, fill deep lesions, and easily remove excess material with irrigation [18,19]. In fact, like T-CD patch, T-C matrix achieved successful hemostasis as intended in all cases.

This study may be underpowered as it was designed to be conducted at a single institution in a short period, and considering the recruitment capacity, it was designed to have 80% statistical power. Risk factor interpretation was limited because the number of cases was not high. A multicenter randomized clinical trial with a large number of patients is needed to clarify the effects of a T-C matrix. Furthermore, there is heterogeneity among the enrolled patients. PD and DP, which have been reported to have differences in POPF rates, were included in this study. Consequently, the sample size in sub-analysis is reduced, and the conclusions may be limited.

Nevertheless, this is the first prospective study to report the efficacy of a flowable hemostatic matrix for the prevention of POPF after pancreatectomy. When the T-C matrix was applied after pancreatectomy, POPF rates were effectively reduced, especially in cases of DP. In addition, the T-C matrix application was a negative risk factor for POPF in this study. In addition, the upper limit of the 95% confidence interval for the difference in the POPF prevention rate between the two groups was −2.83%, which was less than the non-inferiority margin of 22%, thus demonstrating non-inferiority in the efficacy of preventing POPF in the intervention group compared to the control group.

## 5. Conclusions

In conclusion, our findings indicated that flowable thrombin-containing collagen hemostatic matrix (T-C matrix) application is a simple, feasible, and effective method of preventing POPF after pancreatectomy, especially in cases of DP. A larger, randomized controlled trial may be required to confirm the effectiveness of this method.

## Figures and Tables

**Table 1:** Clinicopathological features of patients who underwent pancreatectomy.

Variable	.	Intervention (*n* = 26)	Control (*n* = 27)	*p* Value ^1^
Age (years)	Median, IQR	59 (56~63)	63 (52~70)	0.849
Sex, *n* (%)	Female	14 (53.8%)	12 (44.4%)	0.494
	Male	12 (46.2%)	15 (55.6%)	
Diabetes Mellitus	Yes	2 (7.7%)	7 (25.9%)	0.077
Preoperative Fasting glucose (mg/dL)	Yes	9 (34.6%)	13 (48.1%)	0.406
BMI (kg/m^2^)	Mean, SD	24.2 ± 3.2	22.4 ± 2.4	0.026
ASA classification, *n* (%)	<3	26 (100.0%)	26 (96.3%)	>0.999
	≥3	0 (0.0%)	1 (3.7%)	
Charlson comorbidity score, *n* (%)	<4	13 (50.0%)	9 (33.3%)	0.218
	≥4	13 (50.0%)	18 (66.7%)	
Operative type, *n* (%)	Laparotomy	13 (50.0%)	12 (44.4%)	0.685
	Minimal invasive	13 (50.0%)	15 (55.6%)	
Operative name, *n* (%)	Whipple’s operation	14 (53.8%)	14 (51.9%)	>0.999
	Distal pancreatectomy	12 (46.2%)	13 (48.1%)	
Additional resection, *n* (%)	Yes	6 (22.2%)	3 (11.1%)	0.467
	Gallbladder	0	1	
	SMV or PV	3	2	
	Total gastrectomy/celiac axis	1	0	
	Right hemicolectomy	1	0	
	Liver	1	0	
Operative time, min	Mean, SD	293.5 ± 108.3	280.0 ± 110.9	0.656
Estimated blood loss, *n* (%)	≤400 mL	24 (92.4%)	25 (92.6%)	>0.999
	401~700 mL	1 (3.8%)	1 (3.7%)	
	≥701 mL	1 (3.8%)	1 (3.7%)	
Pathological diagnosis, *n* (%)	Malignancy	15 (57.7%)	11 (40.7%)	0.217
	PDAC	14 (53.8%)	9 (33.3%)	
	Benign	11 (42.3%)	16 (59.3%)	
Pancreas texture, *n* (%)	Soft	12 (46.2%)	15 (55.6%)	0.494
	Firm or hard	14 (53.8%)	12 (44.4%)	
Mass size, cm	Mean, SD	3.4 ± 2.1	3.3 ± 1.7	0.793
Pancreatic duct size, mm	Mean, SD	3.1 ± 1.9	3.0 ± 1.7	0.816
Neoadjuvant therapy, *n* (%)	Yes	4 (15.4%)	1 (3.7%)	0.192
Alternative fistula risk score, *n* (%) ^2^	Low	4 (14.8%)	8 (29.6%)	0.264
	Intermediate	15 (55.6%)	15 (55.6%)	
	High	8 (29.6%)	4 (14.8%)	

^1^ The *p* value was calculated using Student’s *t*-test or Mann–Whitney *U* test for continuous variables and *χ*^2^ test or Fisher’s exact test for binary variables; ^2^ The alternative fistula risk score was determined according to the definition of the Dutch Pancreatic Cancer Group; IQR, interquartile range; ASA classification, American Society of Anesthesiologists physical status classification; SD, standard deviation; BMI, body mass index; PDAC, pancreatic ductal adenocarcinoma; SMV, superior mesenteric vein; PV, portal vein.

**Table 2:** Outcomes of postoperative pancreatic fistula and morbidities for all patients.

Variable	.	Intervention (*n* = 26)	Control (*n* = 27)	*p* Value ^1^
Drain removal, days	Median, IQR	4 (3~5)	5 (3~6)	0.241
POPF, *n* (%) ^2^	Yes	8 (30.8%)	16 (59.3%)	0.037
POPF grade, *n* (%) ^2^	BL	4 (15.4%)	8 (29.6%)	0.438
	B	4 (15.4%)	7 (25.9%)	
	C	0 (0.0%)	1 (3.7%)	
Clinically relevant POPF, *n* (%) ^2^	Yes	4 (15.4%)	8 (29.6%)	0.409
Postoperative complication, *n* (%) ^2^	Yes	9 (34.6%)	17 (63.0%)	0.039
Complication grade, *n* (%) ^2^	≥Grade III	5 (19.2%)	3 (11.1%)	0.444
Length of hospital stay, days	Median, IQR	8 (7~11)	9 (7~14)	0.284
90-day mortality, *n* (%)	Yes	0 (0.0%)	0 (0.0%)	>0.999
Readmission, *n* (%)	Yes	5 (19.2%)	3 (11.1%)	0.409

^1^ The *p* value was calculated using Student’s *t*-test or Mann–Whitney *U* test for continuous variables and *χ*^2^ test or Fisher’s exact test for binary variables; ^2^ Postoperative pancreatic fistula (POPF) and overall complications were assessed and graded based on the criteria of the International Study Group on Pancreatic Surgery (ISGPS) and the Clavien–Dindo complication classification, respectively; IQR, interquartile range; BL, biochemical leakage.

**Table 3:** Sub-analysis of postoperative pancreatic fistula and morbidities for patients who underwent distal pancreatectomy.

Variable	.	Intervention (*n* = 12)	Control (*n* = 13)	*p* Value ^1^
Drain removal, days	Median, IQR	4 (3~5)	4 (3~6)	0.167
POPF, *n* (%) ^2^	Yes	4 (33.3%)	12 (92.3%)	0.004
POPF grade, *n* (%) ^2^	BL	3 (25.0%)	6 (46.2%)	0.018
	B	1 (8.3%)	5 (38.5%)	
	C	0 (0.0%)	1 (7.7%)	
Clinically relevant POPF, *n* (%) ^2^	Yes	1 (8.3%)	6 (46.2%)	0.027
Postoperative complication, *n* (%) ^2^	Yes	4 (33.3%)	12 (92.3%)	0.004
Complication grade, *n* (%) ^2^	≥Grade III	1 (8.3%)	3 (23.1%)	0.593
Length of hospital stay, days	Median, IQR	7 (6–9)	7 (6–14)	0.274
90-day mortality, *n* (%)	Yes	0 (0.0%)	0 (0.0%)	>0.999
Readmission, *n* (%)	Yes	1 (8.3%)	1 (7.7%)	>0.999

^1^ The *p* value was calculated using Student’s *t*-test or Mann–Whitney *U* test for continuous variables and *χ*^2^ test or Fisher’s exact test for binary variables; ^2^, Postoperative pancreatic fistula (POPF) and overall complications were assessed and graded based on the criteria of the International Study Group on Pancreatic Surgery and the Clavien–Dindo complication classification, respectively; IQR, interquartile range; BL, biochemical leakage.

**Table 4:** Univariate and multivariate logistic regression analyses of risk factors for postoperative pancreatic fistula.

Variable	Univariate			Multivariate		
	OR ^1^	95% CI	*p* Value ^2^	OR ^1^	95% CI	*p* Value ^2^
All patients (*n* = 53)						
Intervention			0.031			0.029
Control	3.455	1.119~10.669		4.744	1.172~19.210	
Pancreatic duct size, mm			0.012			0.018
≥3						
<3	6.125	1.501~24.997		7.120	1.399~36.241	
Pancreatic texture			0.002			0.007
Firm or hard						
Soft	7.000	2.088~23.468		6.525	1.668~25.529	
Patients who underwent distal pancreatectomy (*n* = 25)						
Intervention			0.006			0.024
Control	27.000	2.561~284.696		17.379	1.453~207.870	
Pancreatic texture			0.011			0.096
Firm or hard						
Soft	12.000	1.762~81.745		6.666	0.712~62.374	

^1^ The odds ratio (OR) was estimated using a logistic regression model excluding possible confounding variables; ^2^ The *p* value was calculated using a logistic regression model; 95% CI, 95% confidence interval; DP, distal pancreatectomy.
